# Blends of poly(butylene adipate-*co*-terephthalate) (PBAT) and stereocomplex polylactide with improved rheological and mechanical properties

**DOI:** 10.1039/c9ra10827k

**Published:** 2020-03-11

**Authors:** Hongwei Zhao, Huan Liu, Yaqin Liu, Yong Yang

**Affiliations:** School of Material Science and Engineering, Hunan University of Science and Technology Xiangtan 411201 Hunan PR China 1200013@hunst.edu.cn; Hunan Provincial Key Laboratory of Advanced Materials for New Energy Storage and Conversion, Hunan University of Science and Technology Xiangtan 411201 PR China; College of Food Science and Engineering, Hainan University Haikou 570228 PR China yangyong@hainanu.edu.cn

## Abstract

Biodegradable blends of poly(butylene adipate-*co*-terephthalate) (PBAT) and stereocomplex polylactide (sc-PLA) were prepared herein *via* a melt blending method at various sc-PLA loadings. Wide-angle X-ray diffraction and differential scanning calorimetry results verified that complete stereocomplex polylactide crystallites in the PBAT could be achieved. Scanning electron microscopy observation indicated that sc-PLA was dispersed in the PBAT matrix as spherical particles; the dispersed size of the sc-PLA did not display a pronounced increase once the content of sc-crystallites reached a critical gel point. As solid fillers, sc-PLA could reinforce the PBAT matrix in a relatively wider temperature region and accelerate the non-isothermal crystallization of PBAT. The properties of PBAT were greatly improved after blending with sc-PLA, particularly when a percolation network structure of spherical filler had formed in the blends. In addition, all the blends showed higher yield stresses and moduli than those of the neat PBAT but exhibited reduction in elongations in tensile mechanical tests. These results would be interesting to the industrial polymer materials community, and may be of significant use and importance for the wider practical application of PBAT.

## Abstract 2

Schematic representation of formed solid sc-PLA in the PBAT melting during melt processing.

## Graphical abstract

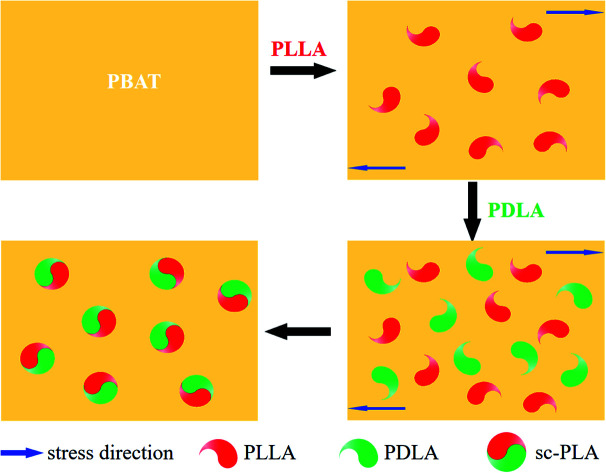

## 1. Introduction

Polymeric materials have been rapidly developed as the most widely used materials over the decades, and made a big contribution to human lifestyle and scientific technology development. However, a series of problems like environmental pollution have become significant challenges for the application of conventional polymeric materials.^1^ In order to solve these problems, intense efforts have been made to promote the development and utilization of biodegradable polymers.^2–4^ Among the biodegradable polymers, poly(butylene adipate-*co*-terephthalate) (PBAT) is an aliphatic aromatic copolyester consisting of two types of comonomer, a rigid butylene terephthalate segment consisting of 1,4-butanediol and terephthalic acid monomers and the flexible butylene adipate section consisting of 1,4-butanediol and adipic acid monomers.^5,6^ PBAT has properties similar to nonbiodegradable polymers like polyethylene with high ductility and low modulus of elasticity.^7^ However, it is worth nothing that low heat resistance, and inferior stiffness are the major shortcomings of PBAT to extend its applications.^8^ In order to broaden its application scope, both physical and chemical modifications based on PBAT have been tried.^9–12^

Poly(lactic acid) (PLA) is derived completely from renewable resources and hence production is sustainable.^13–17^ It also can significantly contribute to the control of green-house gas (CO_2_) emission as a result of carbon capture during plant growth and the eventual complete biodegradability of the PLA matrix. Wang *et al.* who blended PLA and PBAT by solvent-casting methods, obtaining homogeneous films with increased flexibility; this occurs without loss of tensile properties, transparency, or water vapor barrier.^18^ Pan and coworkers prepared the biodegradable PLA/PBAT blends by melt compounding using MDI as chain extender.^19^ It was found that the impact strength of biodegradable PLA and PBAT blends was significantly enhanced successfully by using MDI as chain extender through reactive melt processing. Upon increasing the content of the MDI, the blends showed increased yield tensile strength, modulus, and elongation at break. Jiang and coworkers prepared PLA/PBAT blends by melt blending.^20^ It was found that the PBAT component accelerated the crystallization rate of PLA but had little effect on its final degree of crystallinity. With the increase in PBAT content, the blend showed decreased tensile strength and modulus; however, elongation and toughness were dramatically increased. In addition, with the addition of PBAT, the failure mode changed from brittle fracture of the neat PLA to ductile fracture of the blend. Debonding between the PLA and PBAT domains induced large plastic deformation in PLA matrix ligaments.

Due to the presence of a chiral carbon in the skeletal chain, PLA has two stereoregular enantiomers, poly(l-lactide) (PLLA), and poly(d-lactide) (PDLA). A special crystalline structure termed stereocomplex based on CH_3_⋯C=O interactions of stereoselective van der Waals forces can be formed by blending PLLA and PDLA.^21^ This stereocomplex-PLA (sc-PLA) showed its *T*_m_ at about 230 °C, which was about 50 °C higher than that of pure PLLA or PDLA, so that sc-PLA should accordingly have better thermal and mechanical properties, and higher hydrolytic stability than neat PLLA or PDLA.^22,23^ Moreover, the stereocomplexes could form in its miscible or immiscible blends in melt or solution conditions.^24–27^ Yang and coworkers succeeded in preparing stereocomplex crystallites with high crystallinity. The research demonstrated that exclusive stereocomplex crystallites without the homocrystallites of PLA could be formed at processing temperatures as low as 160 °C, either at equimolar PLLA/PDLA blends or non-equimolar PLLA/PDLA blends.^28,29^ PLLA and PDLA rapid crystallization from the melting state, and the appearance of stereocomplex crystallites of PLLA/PDLA blends prepared was in powder form. The PLA stereocomplex crystallites (sc-PLA) played a similar role like nucleating agent in neat PLLA or PDLA, improved mechanical performances, superior thermal stability, and lower thermal and hydrolytic degradation rates.^30,31^ High potential of PLA stereocomplexes to develop semicrystalline materials with improved performances for longlasting applications.^32–34^ Shi *et al.* blended the PLLA/PBAT system with different PDLA.^35^ The sc-PLA crystallites promoted the rheological properties, especially the complex viscosity of PLLA/PBAT system efficiently, which is due to the reserved sc-PLA crystals acted as crosslinking sites. In our previous study, bioresource-based blends of the P34HB and sc-PLA were prepared. The results demonstrated that exclusive stereocomplex crystallites, without homocrystallites of PLLA or PDLA, could be formed at processing temperatures as low as 175 °C with equimolar PLLA/PDLA in the blends, the rheological and mechanical properties of P34HB were greatly improved after blending with sc-PLA.^36^

Therefore, in this work, we presented a simple method to prepare biodegradable blends through melt blending by PBAT, PLLA and PDLA. The processing temperature was chosen above the melt point of PLLA and PDLA, and much lower than that of sc-PLA. Our results demonstrated that the stereocomplex crystals can be formed *in situ* during melt blending process of PBAT with equimolar PLLA and PDLA, as investigated by wide-angle X-ray diffraction (WAXD) and differential scanning calorimetry (DSC). In addition, the morphology analysis, rheological, non-isothermal crystallization and tensile mechanical behaviors of PBAT and its blends were investigated in detail. These results would be interesting to the polymer materials industrial community, which may be of significant use and importance for the wider practical application of the PBAT.

## 2. Experimental

### 2.1. Materials

PBAT (Ecoflex FBX 1200), was supplied by BASF Corporation (Germany). Poly(45% butylene adipate-*co*-55% butylene terephthalate) with a melt flow index of 3.3–6.6 g per 10 min (at 190 °C), density of 1.25–1.27 g cm^−3^, the weight-average molecular weight of 142 000 g mol^−1^. Poly(l-lactide) (PLLA) (4032D) was a commercial product of NatureWorks LLC (USA). It exhibited a weight-average molecular weight of 207 000 g mol^−1^ and polydispersity of 1.73 as determined by gel permeation chromatography (GPC), d-isomer content of PLLA is 2.0%. Poly(d-lactide) (PDLA) were synthesized by the ring-opening polymerization of d-lactide using tin octanoate as a catalyst. It exhibited a weight-average molecular weight of 110 000 g mol^−1^ and polydispersity of 1.92 as determined by GPC.

### 2.2. Preparation of PBAT/sc-PLA blends

Ternary blends comprising PBAT, PLLA and PDLA were prepared by using a Haake batch intensive mixer (Haake Rheomix 600, Karlsruhe, Germany). The melt compounding was performed at 175 °C and a screw speed of 60 rpm. PBAT and PLLA were first mixed for 4 min, and then PDLA was added and mixed for another 4 min. For comparison, the neat PBAT was subjected to the same mixing treatment so as to have the same thermal history as its blends. The PBAT/sc-PLA weight ratio was kept in the range of 100 : 0 to 70 : 30, including 100 : 0, 90 : 10, 80 : 20 and 70 : 30. The PLLA/PDLA weight ratio of PLA was fixed at 1 : 1. After mixing, all the samples were cut into small pieces and then were hot-pressed at 160 °C for 3 min followed by cold press at room temperature to form the sheets with various thicknesses for characterization. For convenience, neat PBAT and its blends were designated as PBAT and PBAT X in the following discussion, where X representing the sc-PLA content (wt%) of the blend.

### 2.3. Characterizations and measurements

Investigation of the crystallization behavior and melting characteristics of PLLA and its blends was carried out using a TA instruments differential scanning calorimeter (DSC) Q20 with the Universal Analysis 2000. Indium was used for temperature and enthalpy calibration. All operations were performed under nitrogen purge, and the weight of the samples varied between 5 and 8 mg. In the case of non isothermal melt crystallization, the samples were heated from ambient temperature to 160 °C at a heating rate of 100 °C min^−1^, held for 2 min to erase the thermal history, cooled to 0 °C at a cooling rate of 10 °C min^−1^ and then reheated to 240 °C at the same rate to investigate the subsequent melting behavior.

Wide angle X-ray diffraction (WAXD) experiments were performed on a Rigaku model Dmax 2500 X-ray diffractometer. The Cu K_*α*_ radiation (*λ* = 0.15418 nm) source was operated at 40 kV and 200 mA. The samples were first hot-pressed into films with a thickness of around 1.0 mm. All the specimens were heat-treated at 160 °C for 3 min in a convection oven before the test. The scanning angle (2*θ*) covered a range of 5–40° for PBAT/sc-PLA blends at a speed of 3° min^−1^, respectively.

The morphology of the fractured surface, which was prepared under liquid nitrogen was observed using a field emission scanning electron microscopy (ESEM; FEI, Eindhoven, The Netherlands) at an accelerating voltage of 15 kV. The fracture surface was coated with a thin layer of gold before the measurement. The particle size of sc-PLA phase dispersed in PBAT matrix was analyzed using an Image-Pro Plus software. The number-average particle size (*d*_n_) and particle size distribution parameter (*σ*) were calculated from the following equations:^37^1
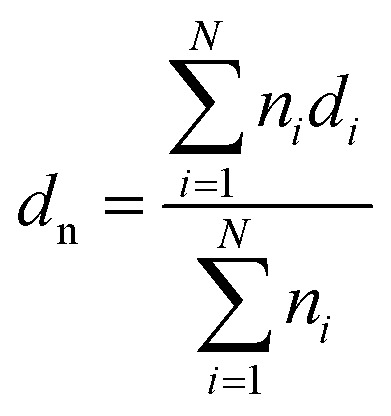
2
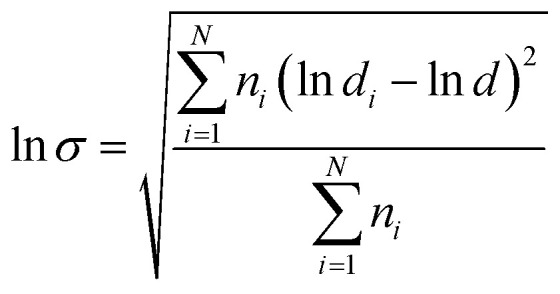
where *n*_*i*_ is the number of sc-PLA particles with the diameter of *d*_*i*_. In the case of monodispersity, *σ* is equal to 1, and when there is polydispersity, *σ* is greater than 1.

Rheological measurements were carried out on a rheometer (AR2000EX, TA Instruments-Waters LLC) equipped with a parallel plate geometry using 20 mm diameter plates. The sheet samples in thickness of 1.0 mm were molten at 160 °C in the fixture and experienced dynamic frequency sweep. The oscillatory frequency swept ranging from 0.1 to 100, with a fixed strain of 1%.

Tensile properties were measured on an Instron-1121 tensile tester in accordance with the standard ISO 527-93 at a crosshead speed of 10 mm min^−1^. The specimens were prepared into dumbbell type with dimensions of 80 mm (total length) × 10 mm (width) × 4 mm (narrow portion width) × 1.0 mm (thickness) by hot compression molding. An average value of five replicated specimens was taken for each composition.

## 3. Results and discussion

### 3.1. Stereocomplex-PLA formation

**Fig. 1:**
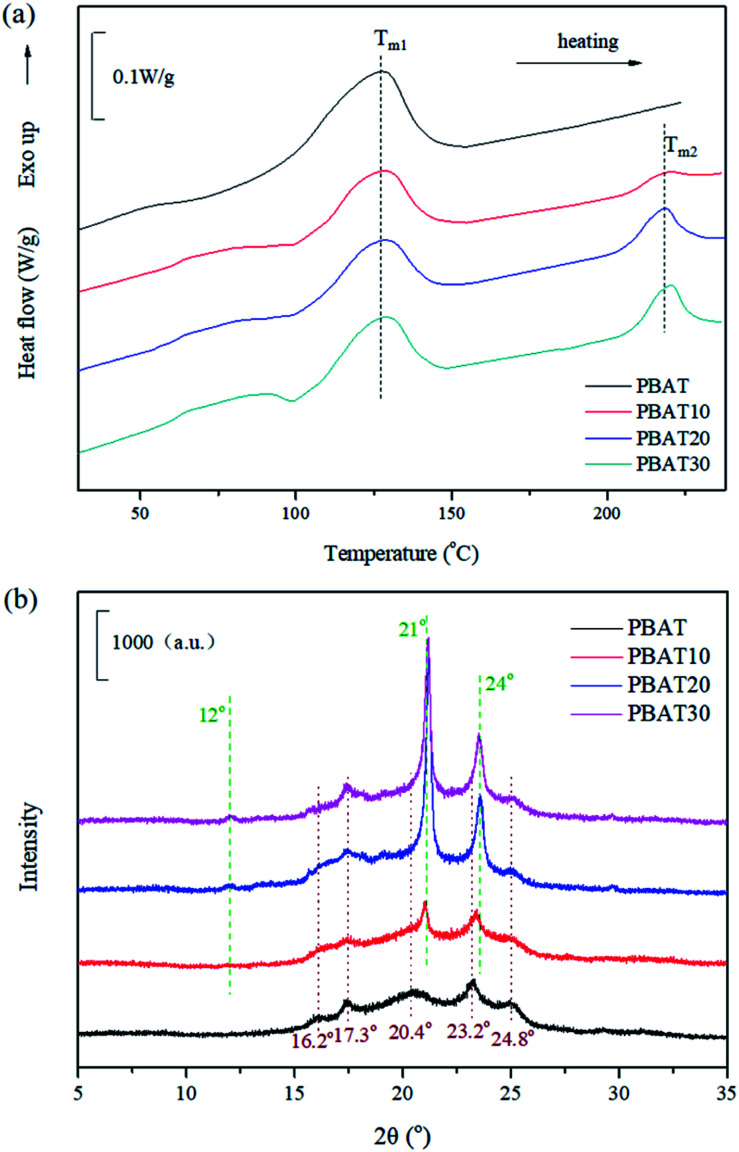
(a) DSC melting and (b) WAXD profiles of the samples.

In order to confirm the formation of stereocomplex PLA in the blends, DSC and WAXD measurements were performed. Fig. 1(a) shows the DSC heating curves at 10 °C min^−1^. During the heating scans, two melting peaks (*T*_m1_ and *T*_m2_) appeared for the blends, indicating the immiscible nature of PBAT and sc-PLA. The melting peaks of PBAT (*T*_m1_) and sc-PLA crystallites (*T*_m2_) at around 127 and 219 °C could be observed for the blends (Table 1). The area of the melting endotherm for the stereocomplex increased as the amounts of PLLA and PDLA in the blend increased. Fig. 1(b) shows the WAXD patterns of the blends containing various content of sc-PLA. All WAXD patterns did not exhibit diffraction peaks of PLLA or PDLA homocrystallites at 2*θ* values of 16, 18.4 and 21.8°,^38^ indicating that no homocrystallites existed. The diffraction peaks of PBAT crystals at 2*θ* = 16.2, 17.3, 20.4, 23.2 and 24.8°.^19^ The most intense peaks of the blended samples were observed at 2*θ* values of 12°, 21°, 24° attributed to (110), (300) and/or (030), (006) planes of the stereocomplex crystals. These peaks were for the stereocomplex crystallized in a triclinic unit cell of dimensions *a* = 0.916 nm, *b* = 0.916 nm, *c* = 0.870 nm, *α* = 109.2°, *β* = 109.2° and *γ* = 109.8°, in which l-lactide and d-lactide segments were packed in a parallel manner, taking helical conformations.^39^ The stereocomplex crystallites could be confirmed by the peaks which became stronger with increasing PLLA and PDLA concentrations.

**Table 1:** Non-isothermal crystallization and melting parameters of neat PBAT and its blends

Samples	*T*_c_ (°C)	*T*_m1_ (°C)	*T*_m2_ (°C)	*t*_1/2_ (min)
PBAT	48.4	127.2	—	2.1
PBAT10	74.5	127.9	219.6	1.5
PBAT20	77.0	127.8	218.8	1.4
PBAT30	79.9	128.0	219.9	1.4

From the aforementioned results of DSC and WAXD, it was confirmed that the stereocomplex could be formed in the blends during melt blending. Zhang and co-workers investigated the nature of the interaction between PLLA and PDLA chains by real-time infrared spectroscopy.^40^ It was found that the interaction between the PLLA/PDLA stereocomplex was ascribed to CH_3_⋯O=C hydrogen bonding. The peak shift of the *ν*(C=O) band occurred during the induction period, which indicated that the CH_3_⋯O=C interaction was the driving force for forming the racemic nucleation of the PLLA/PDLA stereocomplex. Moreover, it is essential for explaining the formation of the stereocomplex during the melt blending based on the influence of the processing temperature window and molecular diffusion since these two factors play important roles in the formation of the stereocomplex. The melting temperature of the stereocomplex crystallites is about 50 °C higher than that of the PLLA or PDLA homocrystallites. Usually, the melt crystallization of a PLLA and PDLA stereomixture is followed by the homocrystallization of PLLA and PDLA along with the crystallization of the stereocomplex. To avoid the effect of the homocrystallization, the melt processing temperature (175 °C), which is between the melting point of pure PLLA or PDLA and that of the PLLA/PDLA stereocomplex is chosen. At the employed processing temperature, the PBAT, PLLA, and PDLA can melt well and only the stereocomplex crystallites can form. The process takes advantage of the processing temperature window below the melting temperature of the stereocomplex crystallites and above that of the homocrystallites of PLLA and PDLA, at which only the stereocomplex crystallites can grow in PBAT melt when the PLLA or PDLA can no longer crystallize.

### 3.2. Non-isothermal crystallization

**Fig. 2:**
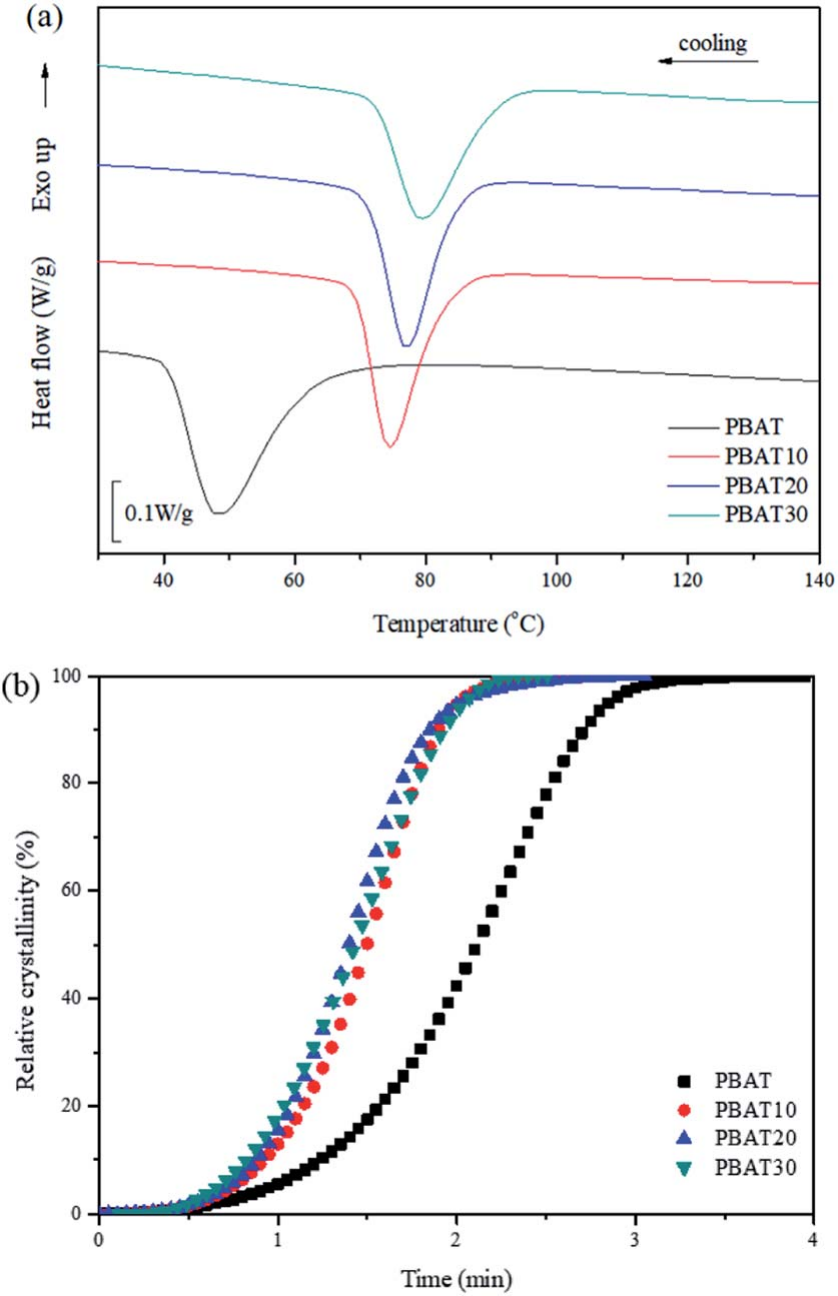
DSC curves of (a) non-isothermal crystallization and (b) conversion curves.

Fig. 2(a) shows the non-isothermal crystallization curves of neat PBAT and its blends. The parameters of interest, such as the crystallization temperature (*T*_c_) and the crystallization half-time *t*_1/2_, which is defined as the half period (*i.e.* 50% crystallization) from the onset of crystallization to the end of crystallization are also summarized and listed in Table 1.

Usually, a high *T*_c_ with a narrow crystallization temperature range observed under cooling conditions reflects a faster crystallization rate. With the addition of 10, 20 and 30% sc-PLA, the crystallization exothermal peak shifts to a higher temperature by *ca.* 26.1, 28.6 and 31.5 °C in comparison with neat PBAT, respectively. In addition, the relative crystallinity (*X*_*t*_) at a given crystallization time (*t*) can be calculated from the integrated area of the DSC curve from *t* = 0 to *t* divided by the whole area of the exothermal peak. The conversion curves of *X*_*t*_*versus t* for neat PBAT and its blends are presented in Fig. 2(b). All of the curves exhibit a sigmoid dependence on time and neat PBAT complete the crystallization within 5 min. It is found that the non-isothermal crystallization of PBAT becomes faster with addition of sc-PLA. These results indicate that formation of sc-PLA particles could accelerate the non-isothermal crystallization of PBAT. However, the more detailed crystallization behaviors of the PBAT/sc-PLA blends remains requires further investigation.

### 3.3. Morphology analysis

**Fig. 3:**
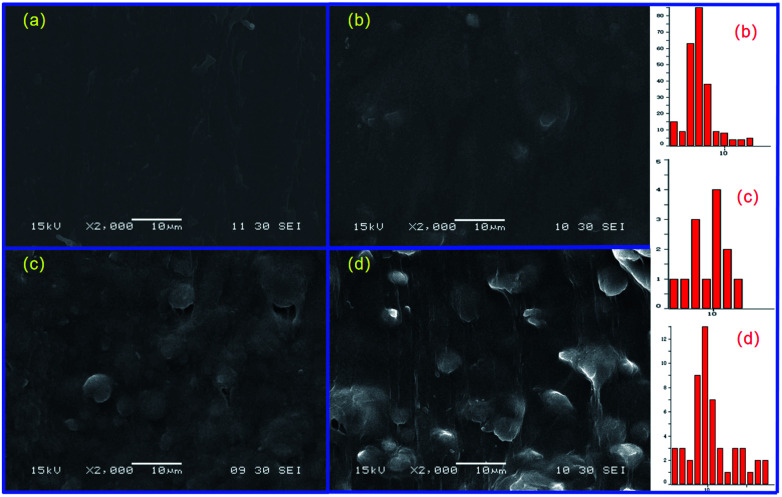
The fracture surfaces of blending samples with various sc-PLA concentrations: (a) neat PBAT, (b) PBAT10, (c) PBAT20, and (d) PBAT30.

**Fig. 4:**
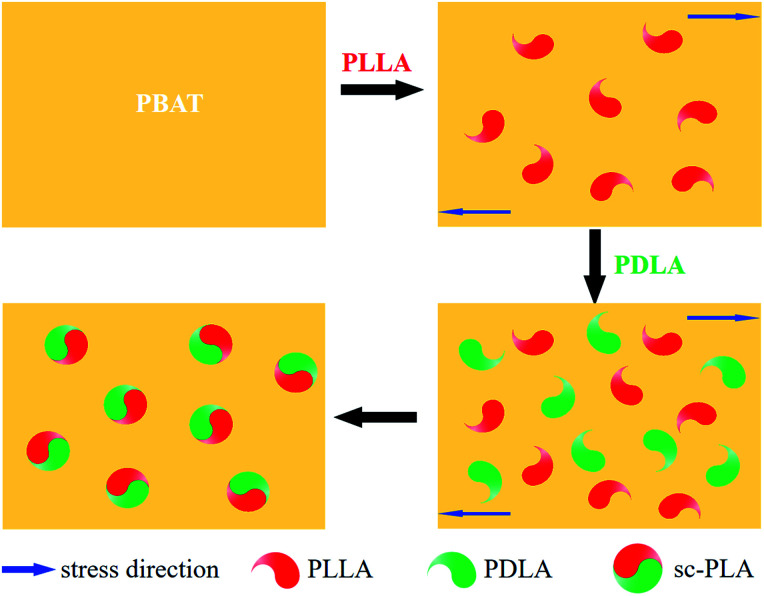
Schematic representation of formed solid sc-PLA in the PBAT melting during melt processing.

Winter and coworkers pointed out that polymer crystallization could be viewed as a physical gelation process and that the transition between liquid-like and solid-like behaviour occurred at a critical gel point.^41,42^ Phase morphology plays a decisive role in determining the physical properties of polymer blend. Accordingly, it is necessary to investigate the phase morphology of the *in situ* formed sc-PLA in the PBAT melt. Fig. 3 shows the fracture surfaces and the particle size distribution diagrams (analyzed by Image-Pro Plus software) of PBAT/sc-PLA blends with various sc-PLA concentrations. For a clear comparison, the statistical results of the sc-PLA particle sizes and their distributions were summarized in Table 2. As shown in the graphs, the fracture surfaces of neat PBAT exhibited a wrinkled structure sc-PLA phase domains dispersed as white dots in the PBAT matrix with distinct interface. The number-average particles size of sc-PLA in PBAT10, PBAT20 and PBAT30 was around 4.36, 7.82 and 8.25 μm, respectively. The dispersed size of the sc-PLA display a pronounced increase with an increase of the PLLA and PDLA contents from 10 to 20 wt%. However, for PBAT20 and PBAT30 blends, the particle size the dispersed size of the sc-PLA did not display a pronounced increase. It should be noted that for many immiscible polymer blending systems, the dispersed phase size tended to increase with an increase in content, which was due to the fact that the main mechanism governing the morphology development of polymer blend was the balance between droplet breakup and coalescence.^43,44^ In our case, once the content of sc-PLA reached a critical gel point, the transition between liquid-like and solid-like behaviors would occur, and the formed solid sc-PLA would be embedded in the PBAT melt. The solid sc-PLA could not be deformed under shear force during melt processing, and the neighboring solid sc-PLA particles could not fuse together. Hence, the dispersed size of the sc-PLA display a content-independence phenomenon once the content of sc-crystallites reached a critical gel point. Schematic representation of formed solid sc-PLA in the PBAT melting during melt processing is illustrated in Fig. 4. However, the exact mechanism of sc-PLA formation and dispersion in the PBAT matrix during melt blending remains unknown and requires further investigation.

**Table 2:** Minimum particle sizes (*d*_min_), maximum particle sizes (*d*_max_), number-average particle sizes (*d*_n_), particle-size distribution parameter (*σ*) and interquartile range for the blends

Samples	*d*_min_ (μm)	*d*_max_ (μm)	*d*_n_ (μm)	*σ*	Interquartile range
PBAT10	1.4	14.0	4.4	1.4	7.1
PBAT20	2.0	16.0	7.8	1.4	8.0
PBAT30	2.0	26.0	8.3	1.6	12.0

### 3.4. Rheological properties and percolation

**Fig. 5:**
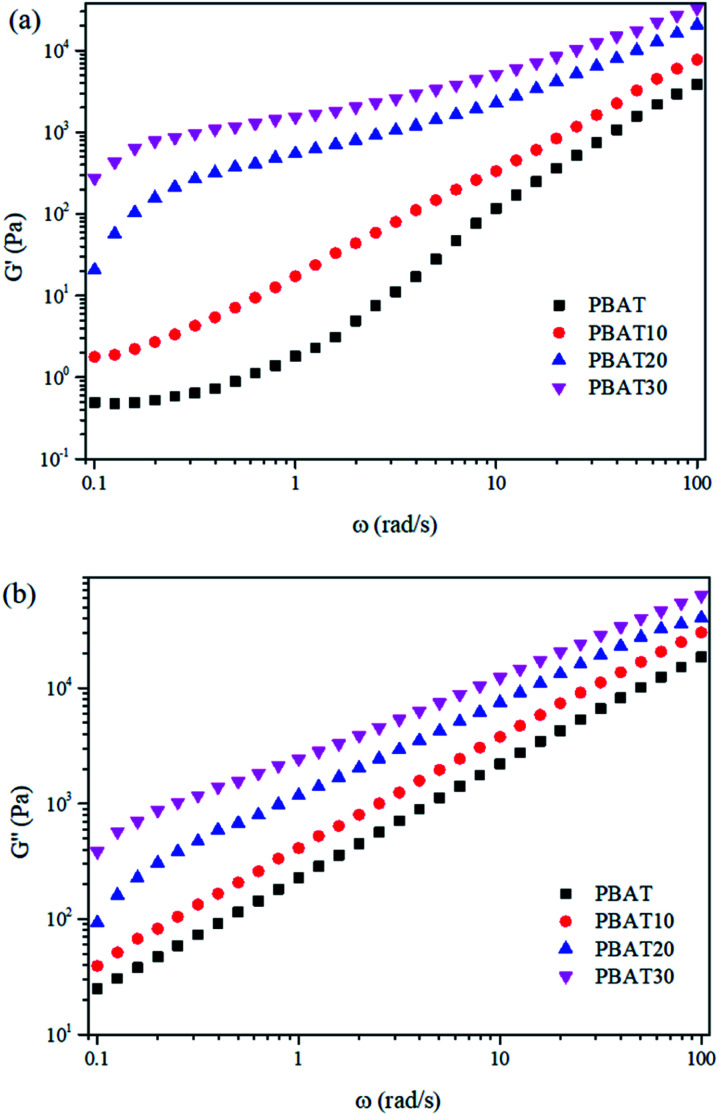
(a) Dynamic storage (*G*′) and (b) loss (*G*′′) modulus for the neat PBAT and its blends.

Dynamic rheological properties are highly sensitive to structure and interactions within the polymer melt. Since the solid structure of a material can be preserved under small-strain test conditions, dynamic rheology testing is often used to evaluate the morphological structure of polymer blends or composites.^45,46^ Due to the characterization temperature far below the melting point of sc-PLA, the sc-PLA phase in the blends could not be melted. Therefore, it is expected that these sc-PLA sphere structures formed *in situ* can reinforce the melt in a relatively wider temperature range. To explore the effect of formed sc-crystallites on the melt rheological properties of the blends, oscillatory shear rheological measurements were carried out at 160 °C. Here, the melting point of PBAT is around 127 °C and that of sc-crystallites is around 218 °C (as shown in Fig. 1(b)); thus, at 160 °C only PBAT were melted, and sc-crystallites were reserved in the melt of the blends. Fig. 5 shows the frequency dependences of storage modulus (*G*′), loss modulus (*G*′′), for neat PBAT and its blends with different concentrations of sc-PLA.

**Fig. 6:**
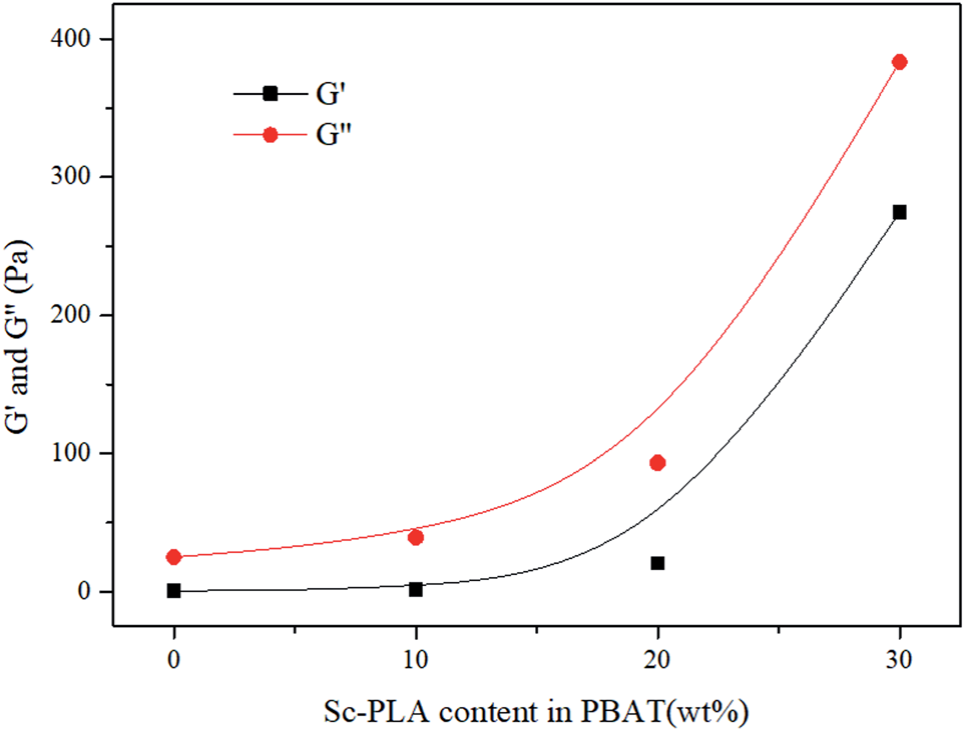
*G*′ and *G*′′ as a function of sc-PLA content in the blends (*ω* = 0.1 rad s^−1^).

**Fig. 7:**
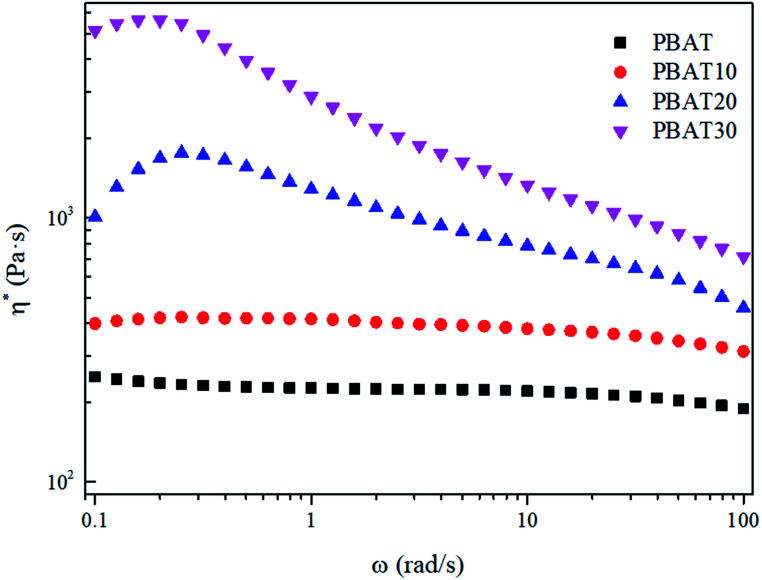
Plots of complex viscosity (η*) *versus* frequency for the neat PBAT and its blends.

In Fig. 5(a) and (b), In the terminal (low frequency) zone, the PBAT melt demonstrated a typical liquid like behavior-logarithmic storage (*G*′) or loss (*G*′′) modulus *versus* logarithmic angular frequency (*ω*) showed a smooth linear relationship, respectively. Both *G*′ and *G*′′ increased with increasing sc-PLA concentration. With 20 wt% sc-PLA, the blends not only exhibited much higher *G*′, *G*′′ and complex viscosity (η*) (Fig. 7) values than neat PBAT, but also demonstrated drastically different terminal behaviors. At low frequencies (0.1–0.3 rad s^−1^), the *G*′ slopes of the blends increased due to the network has been formed in the melt of the blends with increasing content of the reserved sc-crystallites.^47^ The effect of sc-PLA content in the blends could be seen in the insets of Fig. 6, where the increase in *G*′ and *G*′′ with respect to the neat polymer in the terminal (low frequency) zone became phenomenal up to 20 wt% sc-PLA, indicating that the critical sc-PLA content for the percolation threshold had been reached. Clearly, *G*′ and *G*′′ were very sensitive to the formation of sc-PLA percolation at which *G*′ and *G*′′ showed drastic increases.

The complex viscosity (η*) was also employed to reveal the effect of the reserved sc-crystallites on the melt rheological behaviors. It can be seen in Fig. 7 that the typical Newtonian plateau at low frequencies was observed for neat PBAT and the blends with a sc-PLA concentration of 10 wt%. In the terminal zone, the plots of log(η*) *vs.* log(*ω*) changed from a Newtonian (primary) plateau for PBAT to a clear, shear-thinning behavior for the blends. However, for the blends with higher concentration of sc-PLA, the Newtonian plateau disappeared and shear thinning can be observed at low frequencies providing more evidence of elastic behavior due to the solid network structure of the sc-PLA. Furthermore, for PBAT20, a sharp increase of viscosity can be observed. These results indicate that a network has been formed in the melt of the blends with increasing content of the reserved sc-crystallites. The transition from the liquid-like to solid-like viscoelastic behaviors also demonstrates that the long-range polymer chains motion is restrained by the formed sc-crystallite network significantly.

**Fig. 8:**
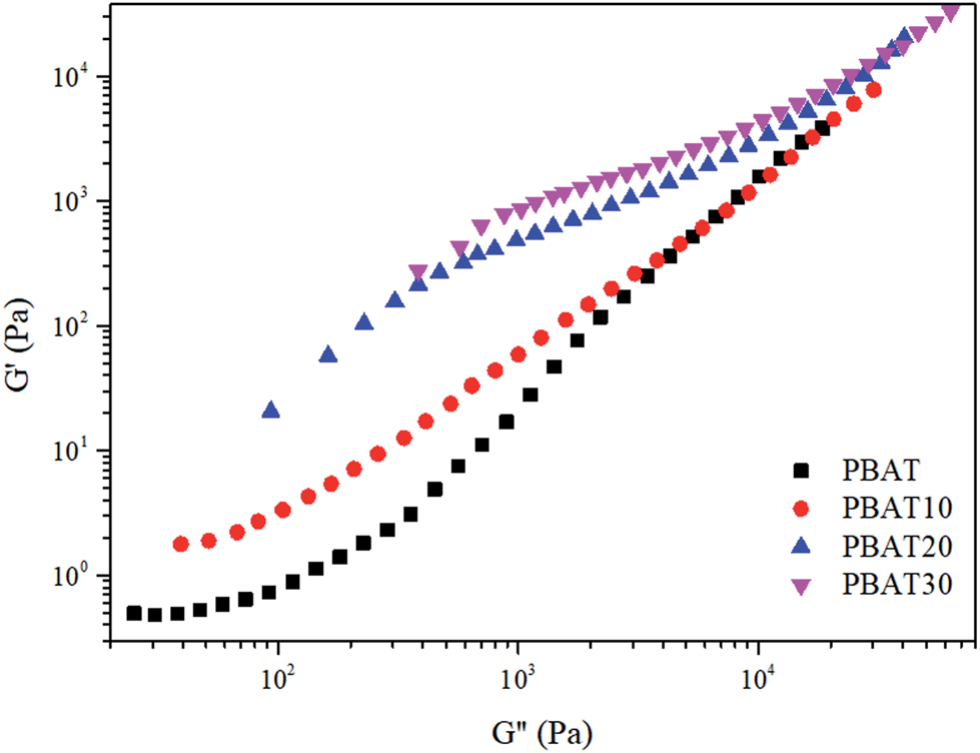
Han plots of dynamic storage modulus (*G*′) *versus* dynamic loss modulus (*G*′′) at 160 °C for neat PBAT and its blends.

As soon as the composites percolated, the large scale polymer chain relaxation behaviors might be highly affected by the percolated sc-PLA network. Fig. 8 shows the Han plots of *G*′ and *G*′′ (ref. 48) for neat PBAT and its composites at 160 °C, respectively. In addition, the reduced slope with the addition of sc-PLA indicated that the composites became more heterogeneous. This indicated that much energy was needed to change the degree of heterogeneity due to the increased physical association within the composites at a high sc-PLA loading. The physical association between sc-PLA and PBAT matrix changed the relaxation behavior of the PBAT chain inevitably. The relaxation time (*k*) could be calculated as follows:^49^3
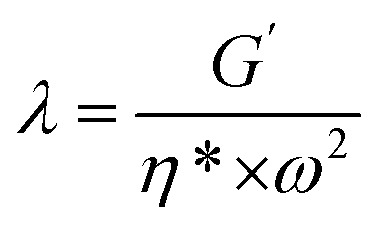


The calculated ratio of the relaxation time of composites to that of neat PBAT (Δ*λ*) increased gradually with sc-PLA loadings and, finally to about 0.3, 1.8 and 5.2 for PBAT10, PBAT20, and PBAT30 samples at the low frequency (0.1 Hz), respectively. This indicated that the role of sc-PLA to make the PBAT chain need longer time for the relaxation became stronger with an increase in sc-PLA loadings. The presence of sc-PLA greatly restricted the chain mobility of PBAT matrix once percolation network structure forms.

**Fig. 9:**
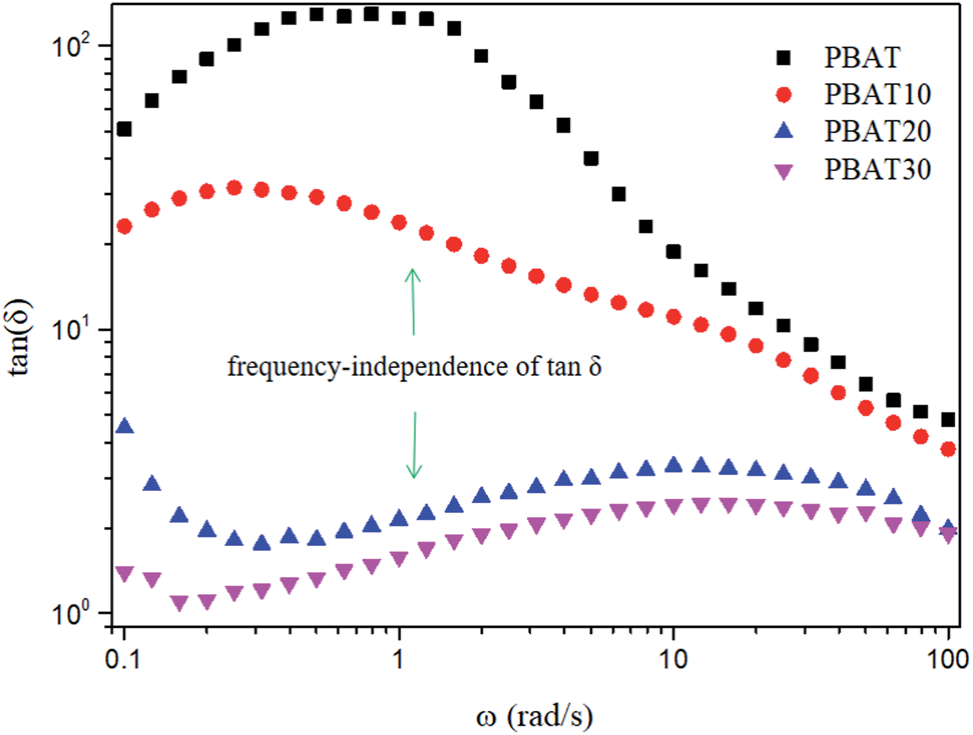
Plots of tan *δ versus* frequency for the neat PBAT and its blends.

The loss tangent (tan *δ* = *G*′′/*G*′) is an essential parameter characterizing the relaxation behavior of the viscoelastic materials and is regarded more sensitive to the relaxation changes than *G*′ and *G*′′.^50^ The frequency dependence of the loss tangent during the gelation process is depicted in Fig. 9. It can be seen in Fig. 9 that tan *δ* of neat PBAT decreased with increasing frequency, which was a typical behavior for viscoelastic liquid. With increasing sc-PLA concentration, tan *δ* decreased gradually, reflecting that the elastic response of the melt became more significant when the formed sc-crystallites increase. When sc-PLA concentration reached 20 wt%, a tan *δ* peak, an indicator of a dominant elastic response of the melt could be observed.^51^ Therefore, it can be inferred that a frequency-independent loss tangent exists between sc-PLA concentration of 10 and 20 wt%, so the percolation threshold should be loaded between 10 and 20 wt% sc-PLA.

**Fig. 10:**
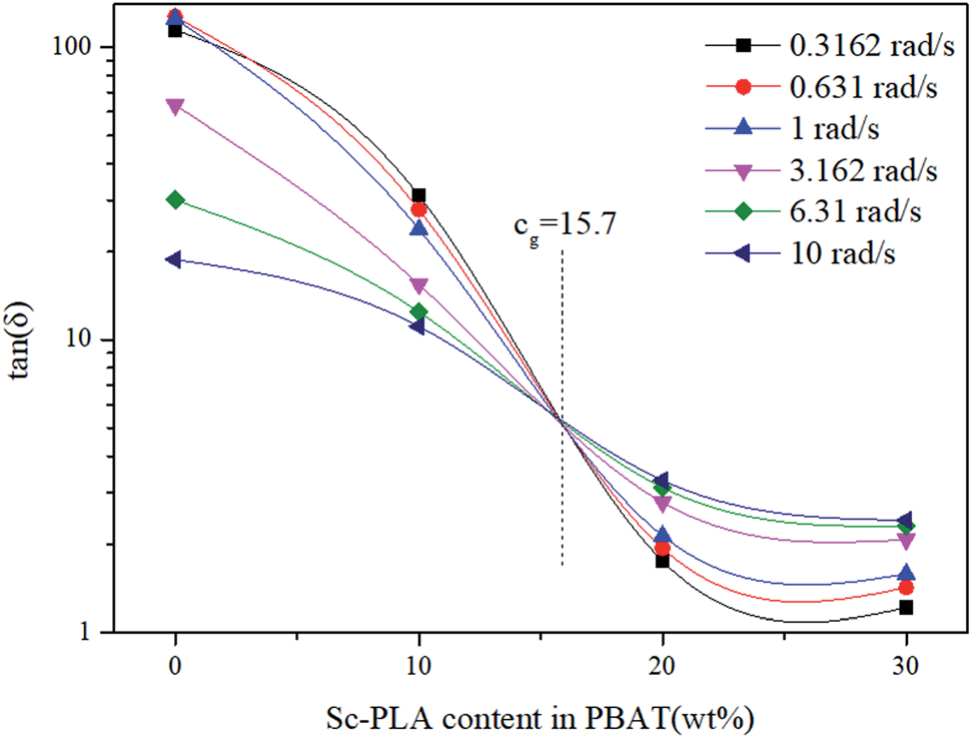
The loss tangent tan *δ* as a function of sc-PLA concentration for the blends.

The frequency-independence of tan *δ* for characterizing gelation, can be applied in an alternative way for determining accurately the gel point without a gelling system exactly at the gel point. Thus, in order to find the percolation threshold, multifrequency (0.3–10 rad s^−1^) values of tan *δ versus* sc-PLA concentration are shown in Fig. 10. The values of tan *δ* steady decreased with increasing sc-PLA concentration. The cross point in this plot resulted in a value of tan *δ* becoming frequency independent at a particular gelation concentration *c*_g_ = 15.7%, which could be defined as the percolation threshold. However, the exact percolation threshold of the PBAT/sc-PLA blends remains requires further investigation.

### 3.5. Tensile mechanical property

**Fig. 11:**
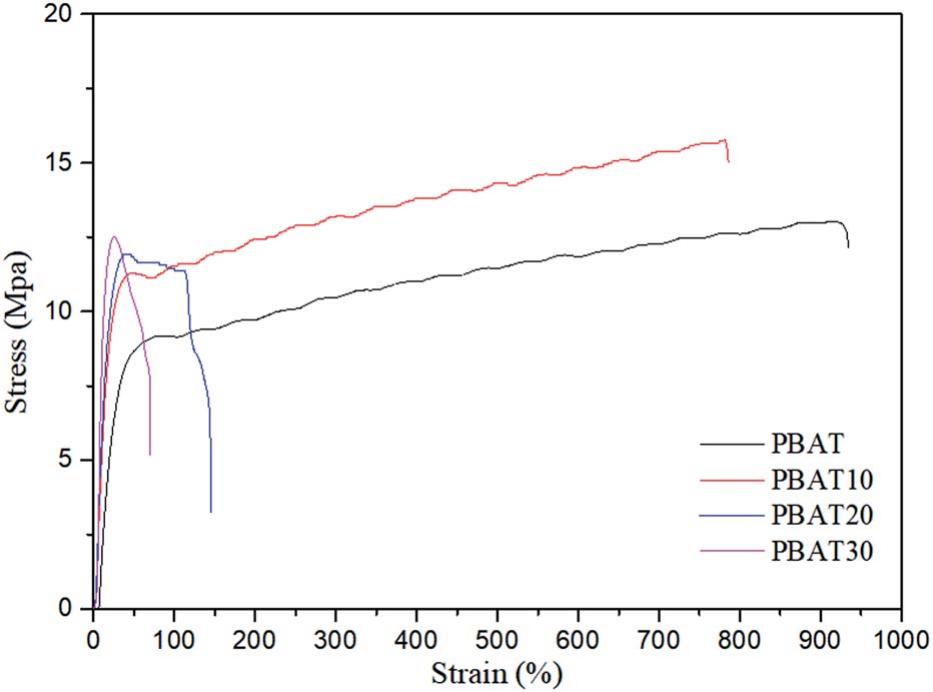
Stress–strain curves of the blends with various sc-PLA contents.

Fig. 11 shows the tensile stress–strain curves of the neat PBAT and PBAT/sc-PLA blends. The neat PBAT behaved like a soft polymer, displaying very low yield stress, modulus, and high ductility. The blends showed typical tensile behavior for a system consisting of a rigid phase dispersed in a soft matrix. The influences of sc-PLA content in the blends on modulus, tensile strength, elongation at break and yield stress of the blends were further compared in Table 3. All the blends showed higher moduli than those of neat PBAT but exhibited reduction in elongations. The increases in modulus were direct consequences of the suppression of the global cooperative chain movement of the PBAT by the percolated sc-PLA network structure. Moreover, when the content of sc-PLA was up to 20 wt%, the modulus of the resulting blends increased dramatically. This phenomenon indicated that the network structure of spherical sc-PLA formed in the blend. Accordingly, the elongation at break also reduced significantly with the formation of sc-PLA network structure. The results indicating that significant reinforcement effect was achieved by adding sc-PLA. The mechanical properties were in good agreement with the variation in the morphological structure of the blends as observed by electron microscopy and rheological tests.

**Table 3:** Static mechanical properties of the neat PBAT and its blends

Samples	Modulus (MPa)	Tensile strength (MPa)	Elongation at break (%)
PBAT	22.8 ± 1.2	9.1 ± 0.8	935.2 ± 23
PBAT10	50.9 ± 0.8	11.2 ± 0.5	785.8 ± 13
PBAT20	57.6 ± 2.4	11.9 ± 0.6	146.9 ± 12
PBAT30	77.5 ± 1.3	12.5 ± 1.2	72.8 ± 9

## 4. Conclusions

Biodegradable PBAT/sc-PLA blends were prepared *via* a melt blending method at various sc-PLA loadings from 10 to 30 wt%. The non-isothermal crystallization, rheological, morphology and mechanical properties of the blends, were investigated. The results demonstrated that exclusive stereocomplex crystallites, without homocrystallites of PLLA or PDLA, could be formed at processing temperatures as low as 175 °C with equimolar PLLA/PDLA in the blends. The formation of sc-PLA particles could accelerate the non-isothermal crystallization of PBAT as a nucleating agent. SEM images indicated that sc-PLA were uniformly dispersed in PBAT matrix. The dispersed size of the sc-PLA formed *in situ* increase with increasing PLLA and PDLA contents and then reach a plateau once the content reached a critical gel point. The rheological behavior of the neat PBAT and PBAT/sc-PLA blends were investigated in detail using various techniques. As the sc-PLA loading reached up to 20 wt%, percolated sc-PLA network structures could be formed. The percolation threshold in rheological results in a value of tan *δ* becoming frequency independent at a particular gelation concentration of 15.7%. In addition, all the blends showed higher yield stresses and moduli than those of the neat PBAT but exhibited reduction in elongations in tensile mechanical test.

## Conflicts of interest

There are no conflicts to declare.

## Supplementary Material
